# Regional variation of medical expenditures attributable to hypertension in China’s middle-aged and elderly population

**DOI:** 10.1097/MD.0000000000032395

**Published:** 2022-12-23

**Authors:** Huilin Sheng, Weihua Dong, YunZhen He, Mengyun Sui, Hongzheng Li, Ziyan Liu, Huiying Wang, Zhi Chen, Long Xue

**Affiliations:** a Suzhou Medical College of Soochow University, Suzhou, China; b Putuo Maternity and Infant Hospital, Shanghai, China; c Jiangxi Provincial People’s Hospital The First Affiliated Hospital of Nanchang Medical College, Jiangxi, China; d School of Public Health, Fudan University, Shanghai, China; e Huashan Hospital, Fudan University, Shanghai, China.

**Keywords:** China, expenditures, hypertension, regional variation

## Abstract

Hypertension is a prevalent and costly health condition in China. Little is known about variation of the inpatient and outpatient expenditures attributable to hypertension between prefecture-level administrative regions (PARs) and the drivers of such variation among China’s middle-aged and elderly population.

**Methods:**

We obtain data from China Health and Retirement Longitudinal Survey between 2011 and 2015, panel tobit models were used in our study to estimate differences across 122 PARs. Expenditure variation was explained by the characteristics of individuals and regions, including measures of healthcare supply.

**Results:**

The cost of treatment for patients with hypertension varies greatly geographically, with the highest outpatient and inpatient costs being 77 and 102 times the lowest, respectively. After adjustment for the individual and PAR character, there are associations between expenditure and region bed density.

**Conclusion:**

There were significant regional differences in the outpatient and inpatient costs of middle-aged and elderly patients with hypertension in China, the difference between individuals may be an important reason, which has little to do with regional economic development differences, but is related to regional bed density.

## 1. Introduction

The prevalence of hypertension in China has increased from 6.7% in 2008^[1]^ to 23.2% in 2015,^[2]^ while the control rate of hypertension (systolic blood pressure ≤ 140 mm Hg and diastolic blood pressure ≤ 90 mm Hg) was less than 15%.^[2]^ If the 2017 American College of Cardiology/American Heart Association Guidelines was adopted, the prevalence of hypertension in China will reach 46.4%, the control rate of hypertension (systolic blood pressure ≤ 130mm Hg and diastolic blood pressure ≤ 80 mm Hg) would be reduced to 3.0%.^[3]^ According to research, 87% of deaths from heart disease, 71% from stroke, and 43% of deaths from chronic kidney disease are related to hypertension in China.^[4]^ It has posed a great economic burden on both patients and society.^[5]^ Based on the China National Health Service survey in 1998 and 2003, the annual medical expenditure on hypertension is 319 billion Chinese Yuan (CNY) ($51 billion),^[6]^ in Guangdong and Hubei provinces in China showed that the average medical expenses of patients with hypertension were 7054 CNY($1133) and 4556 CNY ($731) in 2013, respectively.^[7]^ annual healthcare expenses of patients with hypertension in the United States was on average $2000 higher than those of individuals without hypertension. Overall, healthcare costs associated with hypertension amount to around $131 billion, 2003–2014.^[8]^ Hypertension has become a persistent, serious, and urgent problem.

Regional variation in medical expenditures has represented a popular phenomenon around the world.^[9]^ In the UK, there was a 2.23-fold difference in the cost of insulin treatment for diabetics.^[10]^ In Japan, the regional variations in in-hospital spending were 1.7-fold.^[11]^ Medicare Chronic Conditions Dashboard data showed that in 2017, the difference between the regions with the highest and lowest medical costs per capita for hypertension in the United States reached 2.3 times.^[12]^ Identifying regional differences and their influencing factors will help policymakers address inefficiencies in resource allocation and overuse or underuse, control the unreasonable growth of medical expenses.^[13]^ However, currently available studies have seldom investigated regional variation in medical expenditures of patients with hypertension in China. Therefore, this present study was aimed to explore the regional variation in medical expenditures of hypertension and its influencing factors by employing a nationally representative dataset.

## 2. Methods

### 2.1. Data source and analytic regions

Our data came from 2011, 2013, and 2015 panels of the China Health and Retirement Longitudinal Study (CHARLS), a nationally representative sample of Chinese adults aged 45 and over. The CHARLS national sample was drawn using the stratified 4-stage cluster sampling method. In the first sampling stage, 150 county-level units (rural counties or urban districts) were randomly selected by probability proportional to size from a sampling frame containing all county-level units of mainland China excluding those in Tibet. Within each county-level unit, 3 primary sampling units–administrative villages in rural areas or neighborhoods in urban areas—were randomly selected by probability proportional to size. Within each primary sampling unit, 24 households with members aged 45 or older were randomly selected. The member aged 45 or older and his or her spouse (if present) were interviewed face-to-face by trained interviewers in each household, using a structured questionnaire.^[14]^ Modeled after the Health and Retirement Study in the USA and other related aging surveys in the European countries, CHARLS collected information on the participants’ demographics, socioeconomic status, health status, health insurance, and health care utilization in mainland China. Age 45 was selected as the cutoff age by the CHARLS because it is the minimum retirement age in China.

**Figure 1.**
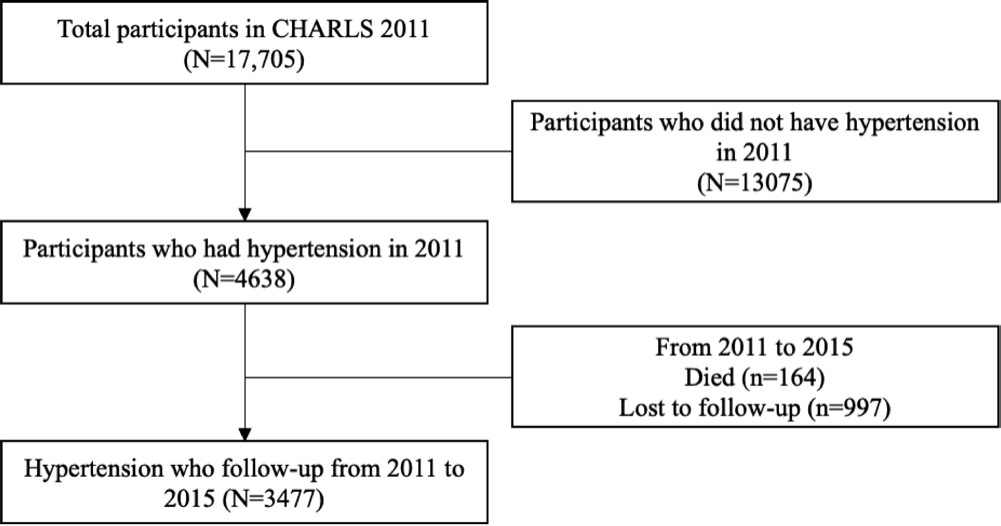
Flow chart of participants.

In 2011, the CHARLS data consisted of 17,705 participants, 4638 of whom self-reported high blood pressure. From 2011 to 2015, 164 hypertensive patients died and 997 were lost to follow-up (669 and 328 were lost to follow-up in 2013 and 2015, respectively). Finally, we included 3433 hypertensive patients for analysis (Fig. 1). We compared the baseline of enrolled with overall hypertensive patients and found no significant difference in variables other than work (see Supplementary Table 1, Supplemental Digital Content, http://links.lww.com/MD/I178), suggesting that the missing data may have less impact on outcome estimates.

A prefecture-level administrative region (PAR) is one of the administrative divisions in China, including prefecture-level municipality, prefectures, autonomous prefectures and leagues. It is a PAR under the jurisdiction of a province or autonomous region. Since November 5, 1983, it has been fixed as administrative division in statistics of national administrative. By the end of 2018, China had 3 leagues, 7 regions, 30 autonomous prefectures, and 293 prefecture-level municipalities among 333 PARs.^[15]^ The CHARLS data includes 122 PARs, but the demographic characteristics are very close to those of the 2010 census, suggesting that the data is well representative of China.^[16]^

### 2.2. Measurement

This study chose the total expenditure of outpatient visits during the last month and inpatient during the last 12 months as our dependent variables. The total expenditure of outpatient visits included both treatment and medication costs, whereas the expense of inpatient consisted of fees paid to the hospital, ward fees but excluding wages paid to a hired nurse, transportation costs, and accommodation costs for yourself or family members.^[17]^ To avoid the impact of outliers, expenses that were above the 99th percentile were set to the 99th percentile value.

The primary independent variable was whether a respondent has been previously diagnosed with hypertension by a doctor or not. Covariates in our study were comprised of age, sex, marital status, overweight, and obesity (defined as body mass index  ≥ 24.0),^[18]^ occupation, annual household income (classified into < 10,000 CNY [1606 US dollar], 10,000–50,000 CNY [1606–8028 US dollar], and ≥ 50,000 CNY [8028 US dollar], using the 2015 USA/RMB exchange rate),^[19]^ and education attainment,^[20]^ self-report health status and self-report diabetes.

Besides, characteristics of regions of residence included GDP per capita, medical consumer price index (MCPI), and healthcare supply. Healthcare supply was measured as physicians and hospital beds per 10,000 inhabitants of the PAR. The MCPI is the average price index of medical care in urban and rural areas of a PAR, which is used to reflect the changes in medical care prices. GDP per capita, MCPI, and healthcare supply capacity was all divided into quintiles based on data from the 2015 wave of CHARLS survey.

### 2.3. Statistical analysis

We computed mean expenses of outpatient visits during the last month and inpatient during the last 12 months for patients with hypertension by PAR. We also plot mean expenses for outpatient and inpatient by regions density of hospital beds. Within each region's density of hospital beds, we further analyzed the distribution of mean expenses by the level of annual household income.

Because more than 50% of the cost of hypertension patients in our sample has a value of 0, and the value that was not 0 basically conforms to the normal distribution, so we did not use the median when describing the sample, but reported the mean value. Given that the skewed distribution nature of cost data (i.e., contain a large number of zero values and) has violated the normal distribution assumption of the ordinary linear model, we turned to a Tobit model to analyze the expenditures.

We firstly fit our data with a multilevel Tobit model and tested the intraclass correlation coefficient. However, the intraclass correlation coefficient was less than 1%, suggesting that cross-regional differences had little impact on costs. Hence, the balanced panel data Tobit model was chosen in our final analysis. Since previous studies using CHARLS suggested that the use of weighting imposed limited impact on regression analyses,^[21]^ our study did not include survey weights in the analysis. All statistical analyses were performed in Stata version 16 (StataCorp LP, College Station, TX).

### 2.4. Ethical approval

All participants provided written informed consent before surveyed and ethical approval for collecting data on human subjects was obtained from the Peking University Institutional Review Board Certificate (IRB00001052-11015).

## 3. Results

### 3.1. Characteristics of hypertension

Table 1 presented the baseline characteristics of patients with hypertension in CHARLS enrolled in 2011. As shown in the table, 55.91% of hypertensive patients were female. The proportion of patients aged 65 and above was 32.99%. More than 80% of hypertensive respondents lived with their partners. Nearly 90% of hypertension were received less than lower secondary education, and only 1.87% had received tertiary education. Close to 95% of respondents have health insurance. Nearly two-thirds of subjects (62.35%) were overweight or obese with body mass index  ≥ 24. Besides, 34.69% of subjects declared that their health status was poor. The proportion of individuals with an annual household income of more than 50,000 CNY ($8028) was 16.82%. 5 (20.88%) respondents living in cities in the lowest quintile of GDP per capita. The results are similar when sampling weights are added.

**Table 1:** Baseline characters of China Health and Retirement Longitudinal Survey samples (% 95%CI).

	Unweighted			Weighted		
	%	95% CI		%	95% CI	
**N**	3477			3477		
**Sex**						
Men	44.09	42.45	45.75	44.17	43.72	44.62
Women	55.91	54.25	57.55	55.83	55.38	56.28
**Age**						
<65	67.01	65.43	68.56	64.54	64.40	64.69
≥64	32.99	31.44	34.57	35.46	35.31	35.60
**Marital status**						
Single	14.32	13.20	15.53	18.89	18.62	19.16
Cohabitant	85.68	84.47	86.80	81.11	80.84	81.38
**Work**						
Not farmer	56.47	54.81	58.11	57.71	57.46	57.96
Farm	43.53	41.89	45.19	42.29	42.04	42.54
**Education**						
Less than lower secondary education	89.39	88.32	90.37	89.51	89.35	89.67
Upper secondary and vocational training	8.74	7.85	9.73	8.33	8.18	8.50
Tertiary education	1.87	1.47	2.38	2.15	2.09	2.22
**Household income**						
<10,000 CNY ($1606)	41.09	39.34	42.86	40.65	40.63	40.67
10,000 CNY–50,000 CNY ($1606–$8028)	42.09	40.33	43.87	42.19	42.17	42.21
≥¥50 000 ($8028)	16.82	15.52	18.20	17.16	17.14	17.18
**Health insurance**						
No	6.49	5.71	7.36	6.86	6.77	6.94
Yes	93.51	92.64	94.29	93.14	93.06	93.23
**BMI (kg/cm**^**2**^)						
<24	37.65	35.97	39.36	38.98	38.70	39.26
≥24	62.35	60.64	64.03	61.02	60.74	61.30
**Self-reported health status**						
Not poor	65.31	63.67	66.92	66.34	66.07	66.60
Poor	34.69	33.08	36.33	33.66	33.40	33.93
**Diabetes**						
without diabetes	86.77	85.60	87.86	86.77	86.57	86.96
with diabetes	13.23	12.14	14.40	13.23	13.04	13.43
**GDP per capita**						
1 (lowest)	20.88	19.55	22.28	21.66	21.62	21.70
2	19.57	18.27	20.93	18.72	18.68	18.76
3	20.65	19.32	22.04	19.73	19.69	19.77
4	19.10	17.81	20.45	17.20	17.16	17.24
5(highest)	19.80	18.50	21.17	22.70	22.66	22.74
**MCPI**						
1 (lowest)	20.32	18.87	21.86	18.83	18.81	18.85
2	21.18	19.70	22.74	21.21	21.19	21.23
3	18.96	17.55	20.46	18.35	18.33	18.37
4	19.89	18.45	21.41	18.40	18.38	18.42
5 (highest)	19.64	18.21	21.16	23.21	23.19	23.23
**Hospital bed per 10,000**						
1 (lowest)	20.45	19.07	21.90	22.09	22.01	22.17
2	21.18	19.78	22.65	20.21	20.13	20.29
3	18.76	17.43	20.16	18.35	18.27	18.43
4	20.93	19.54	22.39	21.98	21.90	22.06
5 (highest)	18.69	17.37	20.10	17.38	17.30	17.46
**Physicians per 10,000**						
1 (lowest)	20.33	18.95	21.78	19.78	19.72	19.84
2	19.78	18.42	21.22	19.35	19.29	19.41
3	20.71	19.33	22.17	18.83	18.77	18.89
4	19.27	17.92	20.69	21.49	21.43	21.55
5 (highest)	19.91	18.55	21.35	20.55	20.49	20.61

BMI = body mass index, MCPI = medical consumer price index.

### 3.2. Cross-regional expenses of patients with hypertension

**Figure 2.**
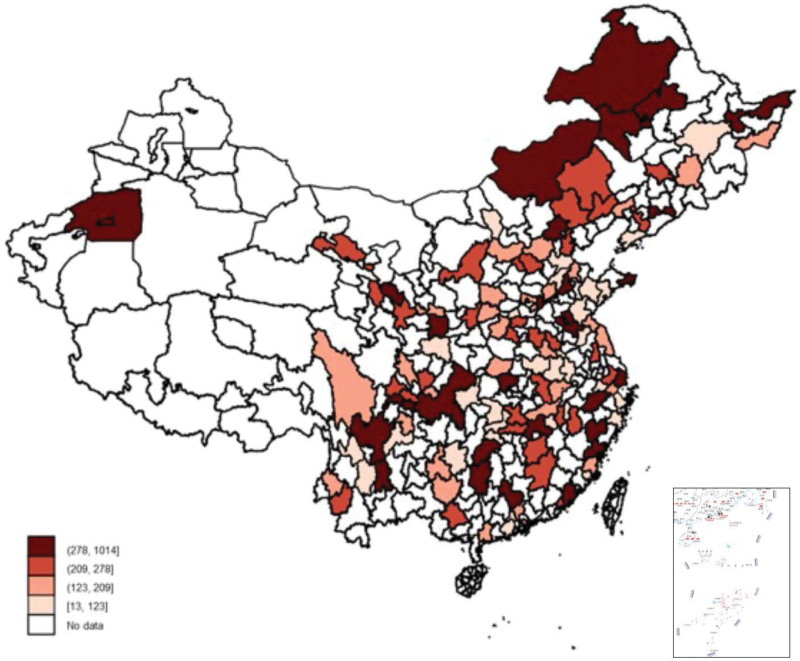
Expenses of outpatient visits of hypertensive patients across PAR. PAR = prefecture-level administrative region.

**Figure 3.**
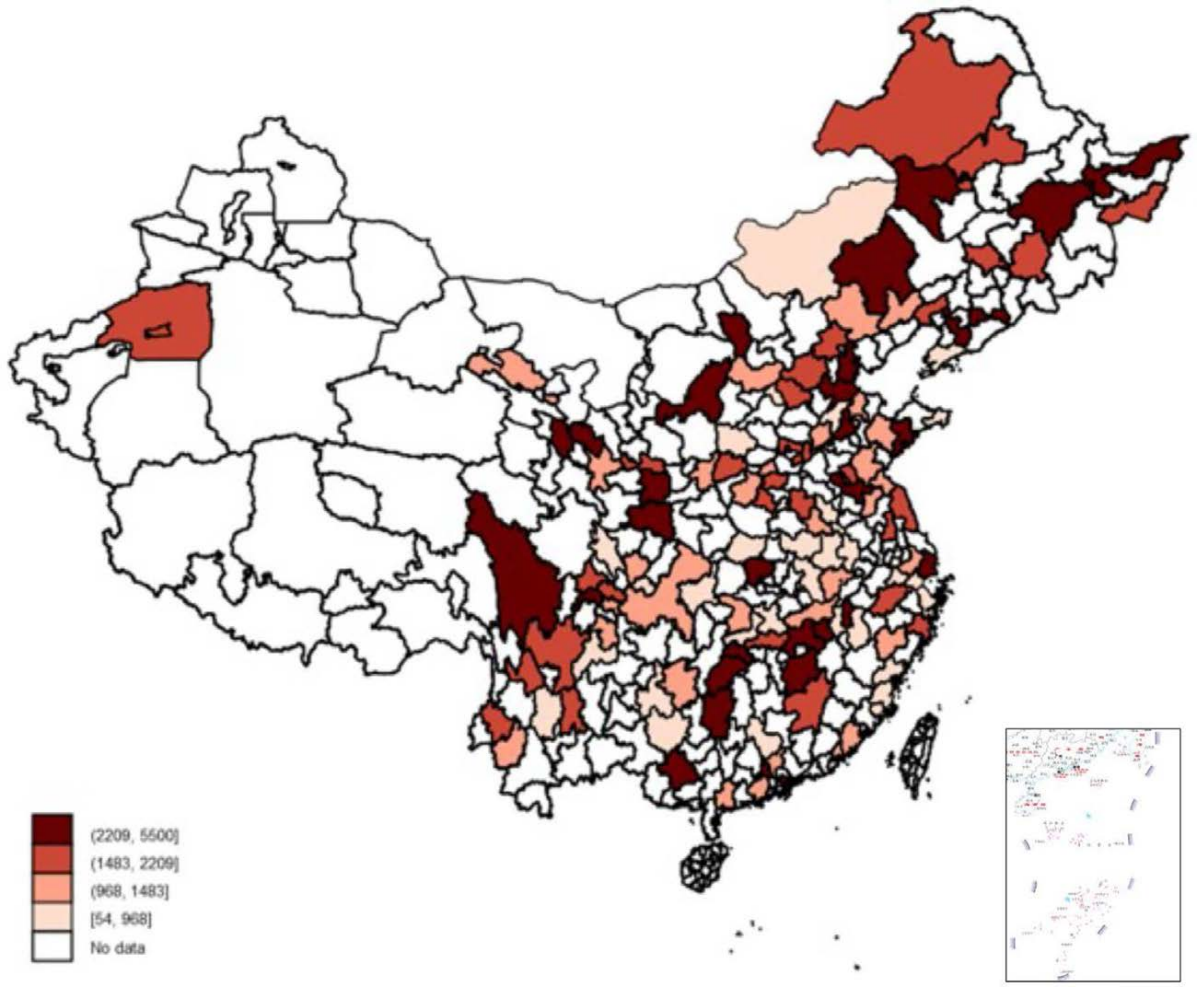
Expenses of inpatient visits of hypertensive patients across PAR. PAR = prefecture-level administrative region.

Figures 2 and 3 showed the mean expenses of outpatient visits during last month and inpatient during the last 12 months by the PAR. There was substantial heterogeneity across regions. In the highest spending region, the expenditure on outpatient and inpatient was 77 and 102 times, respectively, higher than those with the lowest spending regions. We found that the regional differences in both outpatient and inpatient costs were the largest in 2015, followed by 2013, and the smallest in 2011 (Fig. S1 to Fig. S6, Supplementary Digital Content, http://links.lww.com/MD/I179).

**Figure 4.**
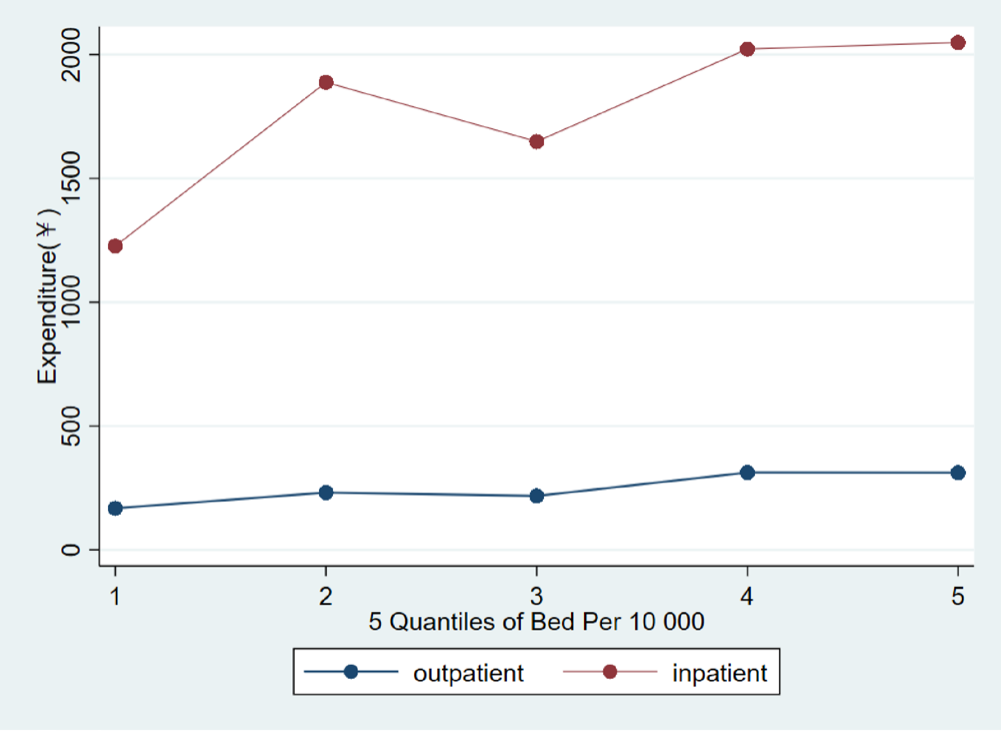
Outpatient and inpatient costs for hypertensive patients in areas with different bed densities.

Figure 4 showed the distribution of costs of outpatient and inpatient for hypertensive patients across the quantiles of hospital beds per 10,000 populations. The mean cost of outpatient in the highest quintile of hospital bed per 10,000 populations was 1.39 times higher than that in the lowest quintile. Similarly, the mean cost of inpatient in the highest quintile of hospital bed density was 1.54 times higher than that in the lowest quantile. Fig. S7 and Fig. S8, Supplementary Digital Content, http://links.lww.com/MD/I180 distribution of expenditure of outpatient and inpatient for hypertensive patients across the quantiles of hospital beds per 10,000 populations in 2011, 2013, and 2015.

### 3.3. The factor of regional variation in expenditure

For expenses of inpatient visits for hypertension patients, age 65 and above, living with a partner, household income greater than 50,000 CNY, poor health status, diabetes, and the density of hospital bed was positively correlated, while female and agricultural work was negatively correlated, as shown in Table 2.

**Table 2:** Tobit models for the expenditure of outpatient and inpatient in hypertension patient.

	**Outpatient expense**		**Inpatient expense**	
	**Coef**	**S.E.**	**Coef**	**S.E.**
**Sex**				
Men	Ref.			
Women	17.79	24.51	−292.11*	168.87
**Age**				
<65	Ref.			
≥65	81.21**	25.35	538.26**	170.47
**Marital status**				
Single	Ref.			
Cohabitant	65.04*	35.12	722.95**	238.74
**Education**				
Less than lower secondary education	Ref.			
Upper secondary and vocational training	30.02	43.53	437.43	298.57
Tertiary education	32.48	97.67	674.48	651.39
**Occupation**				
Not farmer	Ref.			
Farmer	−81.45**	25.31	−791.79***	166.74
**Household income**				
<10,000 CNY ($1606)	Ref.			
10,000 CNY–50,000 CNY ($1606–$8028)	33.43	30.74	157.85	197.77
≥¥50 000 ($8028)	68.39**	28.98	317.23*	186.62
**Health insurance**				
Not covered by health insurance	Ref.			
Covered in health insurance	53.69	55.69	193.42	358.12
**Self-report health status**				
Not poor	Ref.			
Poor	230.72***	26.14	1404.39***	170.43
**BMI**				
<24	Ref.			
≥24	17.75	24.81	87.05	166.15
**Diabetes**				
Without diabetes	Ref.			
With diabetes	46.92	32.16	876.19***	216.97
**GDP per capita**				
1 (lowest)	Ref.			
2	−2.81	36.71	−207.19	235.37
3	−11.32	36.69	75.5	241.48
4	7.15	47.61	156.84	311.95
5 (highest)	−22.57	48.78	392.22	327.25
**MCPI**				
1 (lowest)	Ref.			
2	64.77	56.92	−185.52	366.15
3	36.96	41.43	−251.37	263.44
4	72.80*	40.46	−7.47	258.01
5 (highest)	46.73	37.86	−130.04	242.45
**Hospital bed per 10,000**				
1 (lowest)	Ref.			
2	88.70**	35.33	991.55***	226.44
3	69.25*	36.19	337.23	233.55
4	100.99**	44.16	531.55*	286.06
5 (highest)	67.79	45.84	333.87	301.35
**Physicians per 10,000**				
1 (lowest)	Ref.			
2	−30.44	34.77	159.99	225.95
3	−29.37	45.73	273.58	300.37
4	1.05	47.15	−44.00	311.84
5 (highest)	42.88	50.6	209.99	338.53

* *P* < .1.

** *P* < .05.

*** *P* < .01.

BMI = body mass index, MCPI = medical consumer price index.

The model showed that after adjusting for individual character and region factors, the inpatient expenses of hypertension living in the region with the second highest bed density were expected 991.55 CNY higher on average than those in the region with the lowest hospital bed density, and actual 615 CNY higher on average. However, for patients with inpatient expenses greater than 0, the actual expenses were 435.1 CNY higher on average.

The inpatient expenses of hypertensive patients living in the region with the fourth highest bed density were expected 531.55 CNY higher on average than those in the region with the lowest hospital bed density, and actual 321 CNY higher on average, while the inpatient expenses of patients with higher than zero were actual 227.57 CNY higher on average.

For the outpatient expenses of hypertensive patients, 65 and above age, live with a partner, household-income greater than 50 000 CNY, poor health status, and the density of bed was positively correlated, while agricultural work was negatively correlated, as shown in Table 2..

The model showed that after adjusting for individual character and region factors, the density of beds increased to the second, third, and fourth grades, respectively, the expected outpatient cost of patients with hypertension increased by an average of 64.77, 36.96, and 72.8 CNY, respectively, the actual outpatient cost increased by an average of 52.68, 40.81 and 60.28 CNY, respectively, in hypertensive with outpatient expenses greater than 0, the actual outpatient expenses increased by 37.32, 28.93 and 42.68 CNY on average.

## 4. Discussions

We found large geographic variations in outpatient and inpatient costs for patients with hypertension. In the PAR with the highest expenditure, outpatient and inpatient expenses were 77 times and 102 times higher, respectively, than those in the regions with the lowest expenditure, and the gap is widening, which may be due to the following reasons: because of the severity of the complications of hypertension, we report that the cost of hypertension varies greatly; we use survey data and are affected by patient recall bias. More than 50% of the medical expenses of hypertensive patients in our sample were recorded as zero, which may be related to the low attention paid to the treatment of hypertension in China. The study found that only 30% to 40% of hypertensive patients in China are treated.^[3,22]^

Age ≥ 65, household income greater than 50 000 CNY, poor health status were the most important individual factor associated with the inpatient and outpatient expenditure in hypertension. The differences in economic level between regions were no association with spending of inpatient and outpatient. Measures of healthcare supply showed the density of beds was associated with outpatient and inpatient expenditure.

Baseline profiles of patients with hypertension included in our study were not significantly different from those of several other studies using the CHARLS database.^[23,24]^ Compared with a study of hypertension in the USA,^[8]^ hypertensive patient in the USA was a lower percentage of hypertension in the female (51.5% vs 55.91%), insurance (92.2% vs 93.51%), but the higher percentage in tertiary education (50.8% vs 1.87%). Identified age 65 or older, higher education, and insurance as contributing factors to the medical costs of hypertensive patients, whereas higher income was negatively correlated in the American study,^[8]^ but positively correlated in our study. This may be because rich people in developed countries pay more attention to disease prevention, while in developing countries, rich people in China spend more on medical treatment of diseases. Among patient with hypertension, inpatient’s expenditure negative associate with the female, This could be related to the fact that women had been more likely to use diagnostic and preventive care than men,^[25]^ as a result, they may make more use of outpatient services, better compliance and healthier lifestyle habits,^[1]^ which may reduce or delay the incidence of hypertension complications and thus reduce hospitalization costs.^[26]^ We found the same result that there was a significant negative correlation between women and hospitalization costs.

According to a report by the Centers for Disease Control and Prevention indicated that one-fourth of adults did not have insurance coverage and that they were 7 times more likely to forgo seeing a doctor than those with health insurance,^[27]^ this may be the reason why the medical expenses and insurance related to hypertension patients in the United States. However, our results show that health insurance was not a factor contributing to the expenditure difference, it may be related to the fact that 93.51% of the respondents in this study have medical insurance, which proves that widespread coverage of health insurance throughout the country.

Among the socioeconomic indicators included in our study, both costs of inpatient and outpatient were significantly negatively correlated with agricultural work and positively correlated with high household income. Previous studies have shown that in rural China, low household income hypertension was less likely to be aware of, treated, and controlled for hypertension,^[3,28]^ these factors may contribute to the relationship between socioeconomic factors and medical costs in hypertensive patients in our study.

Regional economic development level is independent of medical costs for people,^[29,30]^ our study also showed an insignificant association between hypertension and geographic economic level. It may be related to the subsidies for hypertension and other chronic diseases in China,^[23]^ in particular, after the implementation of the National Essential Public Health Services Policy program, many researchers reported that the differences in the hypertension patients in rich and poor areas in China were gradually narrowing.^[23,31,32]^

Among healthcare supply indicators we used, the density of doctors was not associated with inpatient and outpatient spending of hypertensive patients, but the density of beds was related. Previous studies on whether the cost of various diseases was related to healthcare supply have shown inconsistent results,^[11,29,30,33,34]^ a Swiss study showed that a fee-for-service system in which suppliers may respond quickly to meet high demand, thus making the effect of doctor density weaker than that in studies from other countries,^[30]^ it may also be the reason why the relationship between doctor density and medical costs in our study was insignificant.

There are several limitations to our study. First, CHARLS are self-reported and subject to recall biases. Second, Previous reports showed that approximately 20% of respondents are unaware that they have hypertension,^[35]^ therefore, the count of hypertension and their expenditure was likely underestimated. Third, our data only included hypertension in the middle-aged and elderly population, and it did not represent the whole hypertensive in China. Fourth, we did not calculate the out-of-pocket for hypertensive patients, which may understate medical costs. Finally, the medical expenses of the hypertensive patients we included in the study did not distinguish the medical expenses of the patients for what reason, which means that we may have included the costs of the patients for diseases not related to hypertension in the study, which may overestimate.

## 5. Conclusions

Our research may be China’s first based on PAR of hypertension patients with medical expenses differences research. We found that a large and growing regional gap between outpatient and inpatient costs for patients with hypertension, the cost of medical care for hypertensive patients may be a positive correlation to age≥65, live with a partner, household income≥¥50,000, self-report poor health status, the density of local beds, which is important for policymakers to the current hot spot in China the blind expansion of hospital size.

## Acknowledgments

We thank the China Health and Retirement Longitudinal Study (CHARLS) team for providing data. Thanks to all the members of the NHC key laboratory of health technology assessment for providing help.

## Author contributions

**Conceptualization:** Huilin Sheng, Weihua Dong, Zhi Chen, Long Xue.

**Data curation:** Huilin Sheng, Weihua Dong, Yunzhen He, Mengyun Sui, Hongzheng Li, Ziyan Liu, Long Xue.

**Formal analysis:** Yunzhen He, Long Xue, Weijian Dong.

**Funding acquisition:** Zhi Chen.

**Investigation:** Yunzhen He, Long Xue.

**Methodology:** Huilin Sheng, Weihua Dong, Mengyun Sui, Huiying Wang, Zhi Chen, Long Xue.

**Software:** Mengyun Sui, Hongzheng Li, Ziyan Liu, Zhi Chen, Long Xue.

**Supervision:** Huiying Wang.

**Validation:** Mengyun Sui.

**Writing—original draft:** Huilin Sheng, Weihua Dong, Long Xue.

**Writing—review and editing:** Huilin Sheng, Yunzhen He, Zhi Chen.

## Supplementary Material
