# Supplementary material for: Regional variation of medical expenditures attributable to hypertension in China’s middle-aged and elderly population
Source: Medicine (Baltimore). 2022 Dec 23;101(51):e32395. doi: 10.1097/MD.0000000000032395 (PMC9794296; doi:10.1097/MD.0000000000032395)
Supplement: Supplementary file 1 [file medi-101-e32395-s001.pdf]

Table S1 Baseline comparison of included and overall hypertensive patients

N	3477		4638		χ2	p
**Sex**						
Men	1533	44.09	2103	45.34	1.301	0.254
Women	1944	55.91	2535	54.66		
**Age**						
<65	2330	67.01	2951	63.63	2.469	0.116
≥65	1147	32.99	1687	36.37		
**Marital status**						
Single	498	14.32	737	15.89	3.786	0.052
Cohabitant	2979	85.68	3901	84.11		
**Occupation**						
Not farmer	1951	56.47	2838	61.68	22.247	0.000
Farm	1514	43.53	1763	38.32		
**Education**						
Less than lower secondary education	3108	89.39	4062	87.58	7.368	0.025
Upper secondary &vocational training	304	8.74	452	9.75		
Tertiary education	65	1.87	120	2.59		
**Household-Income**						
<10,000 CNY ($1,606)	1229	41.09	1552	33.46	3.786	0.151
10,000 CNY - 50,000 CNY ($1,606 - $8,028)	1259	42.09	1651	35.60		
≥¥50 000 ($8028)	503	16.82	727	15.67		
**Health insurance**						
No	224	6.49	324	6.99	0.948	0.330
Yes	3229	93.51	4279	92.26		
**BMI (kg/cm2)**						
<24	1180	37.65	1542	33.25	0.104	0.747
≥24	1954	62.35	2594	55.93		
**Self-reported health status**						
Not poor	2156	65.31	2835	61.13	0.315	0.575
Poor	1206	34.69	1547	33.35		
**Diabetes**						
Without diabetes	2991	86.77	3978	85.77	0.111	0.739
With diabetes	460	13.23	620	13.37		
**GDP per capita**						
1(lowest)	713	20.88	713	20.88		
2	668	19.57	668	19.57		
3	705	20.65	705	20.65		
4	652	19.10	652	19.10		
5(highest)	676	19.80	676	19.80		

**MCPI**						
1(lowest)	568	20.32	568	20.32		
2	592	21.18	592	21.18		
3	530	18.96	530	18.96		
4	556	19.89	556	19.89		
5(highest)	549	19.64	549	19.64		
**Hospital bed per 10,000**						
1(lowest)	641	20.45	641	20.45		
2	664	21.18	664	21.18		
3	588	18.76	588	18.76		
4	656	20.93	656	20.93		
5(highest)	586	18.69	586	18.69		
**Physicians per 10,000**						
1(lowest)	634	20.33	634	20.33		
2	617	19.78	617	19.78		
3	646	20.71	646	20.71		
4	601	19.27	601	19.27		
5(highest)	621	19.91	621	19.91		
