# Supplementary material for: Regional variation of medical expenditures attributable to hypertension in China’s middle-aged and elderly population
Source: Medicine (Baltimore). 2022 Dec 23;101(51):e32395. doi: 10.1097/MD.0000000000032395 (PMC9794296; doi:10.1097/MD.0000000000032395)
Supplement: Supplementary file 2 [file medi-101-e32395-s002.pdf]

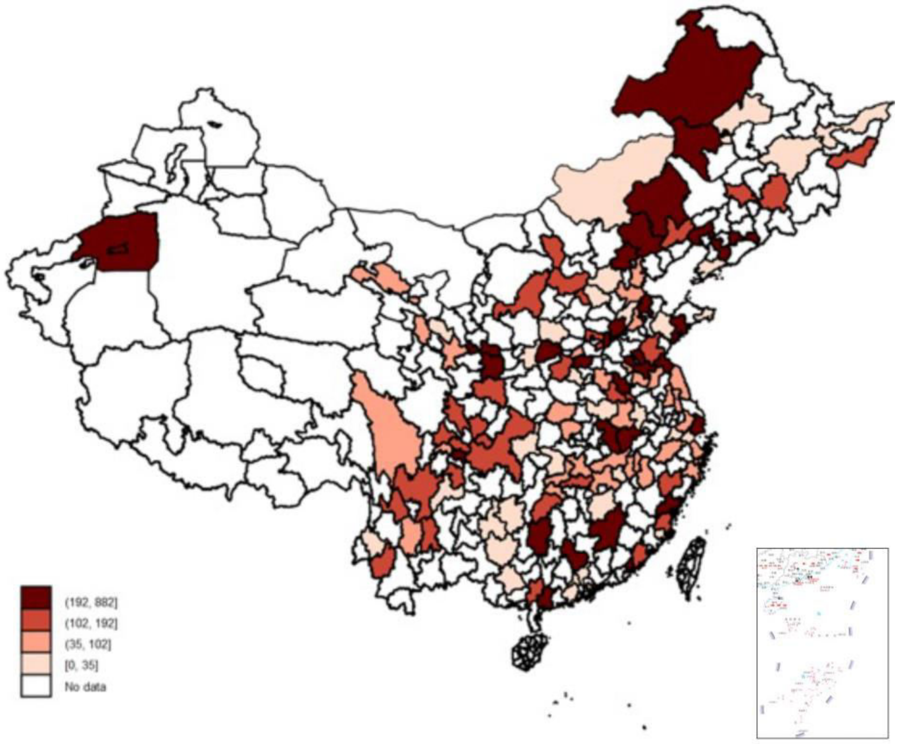

Fig. S1. Expenses of Outpatient of Hypertensive Patients Across PAR in 2011

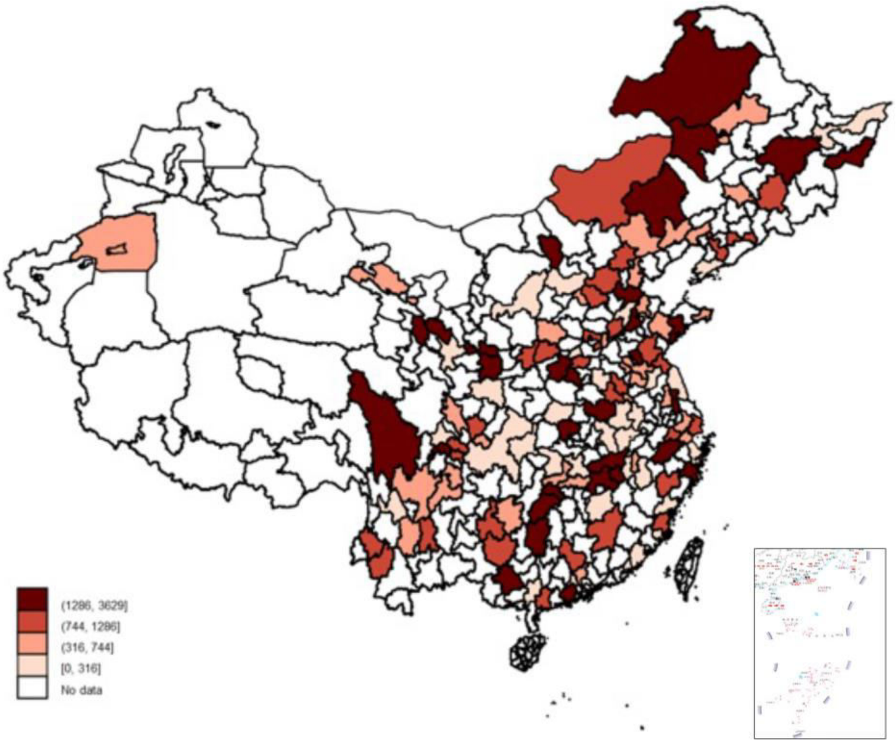

Fig. S2. Expenses of Outpatient of Hypertensive Patients Across PAR in 2013

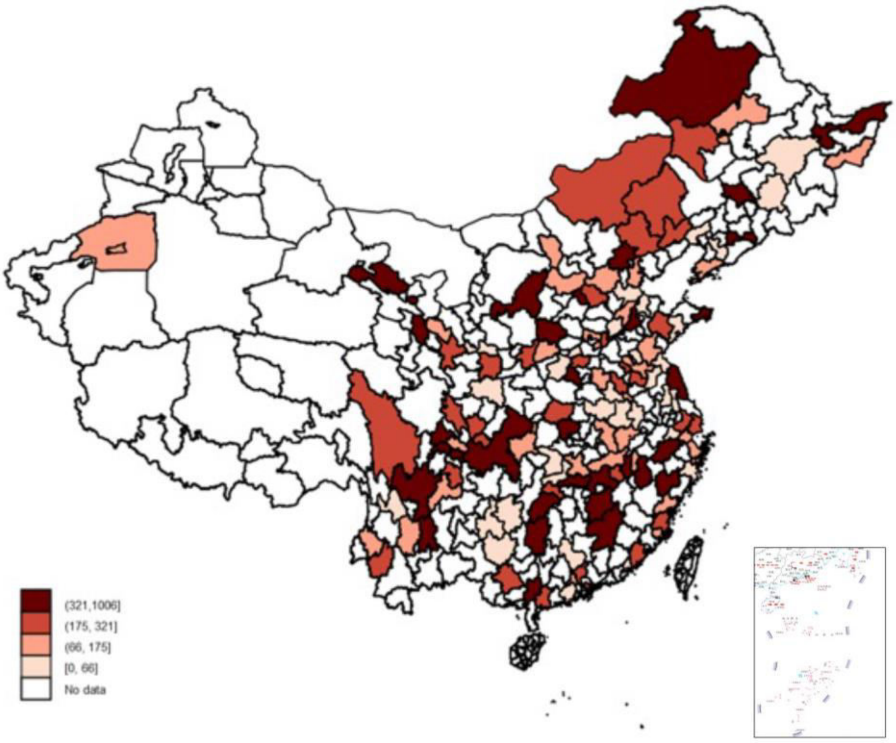

Fig. S3. Expenses of Outpatient of Hypertensive Patients Across PAR in 2015

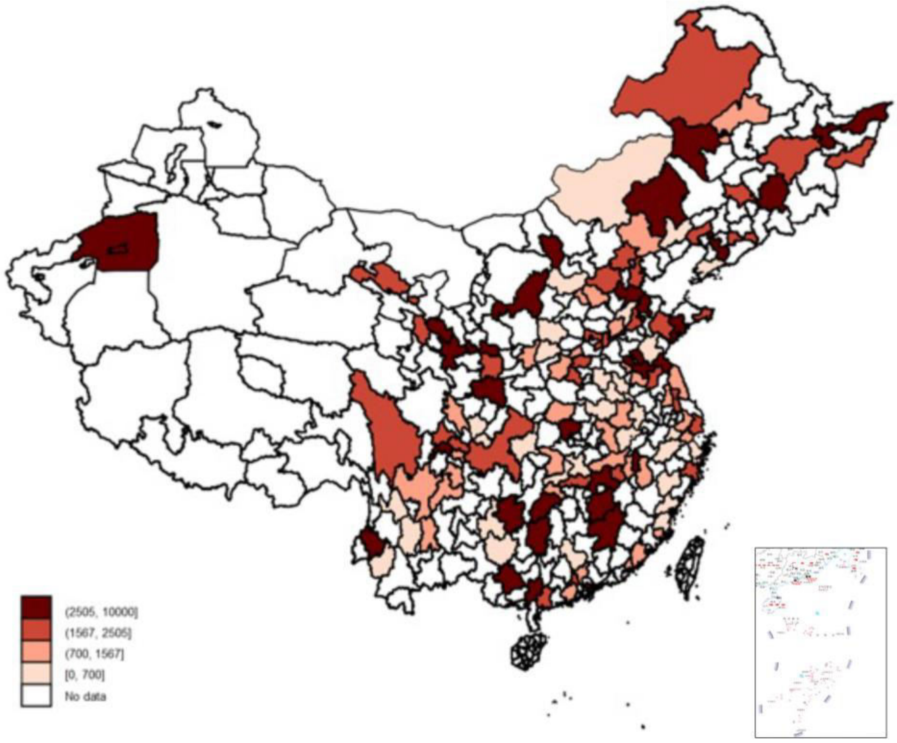

Fig. S4. Expenses of Inpatient of Hypertensive Patients Across PAR in 2011

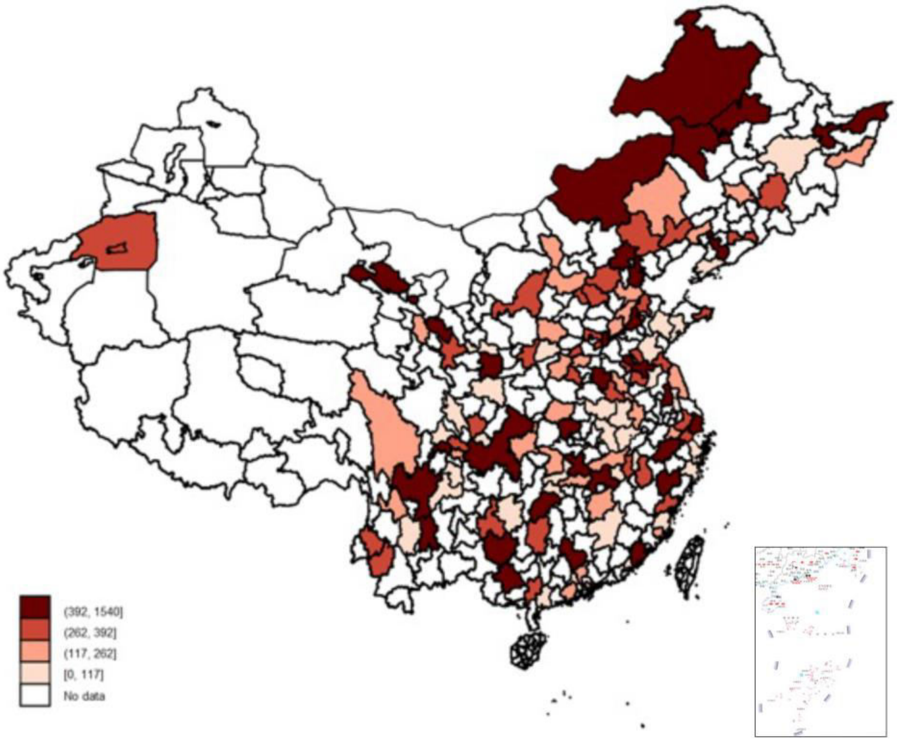

Fig. S5. Expenses of Inpatient Visits of Hypertensive Patients Across PAR in 2013

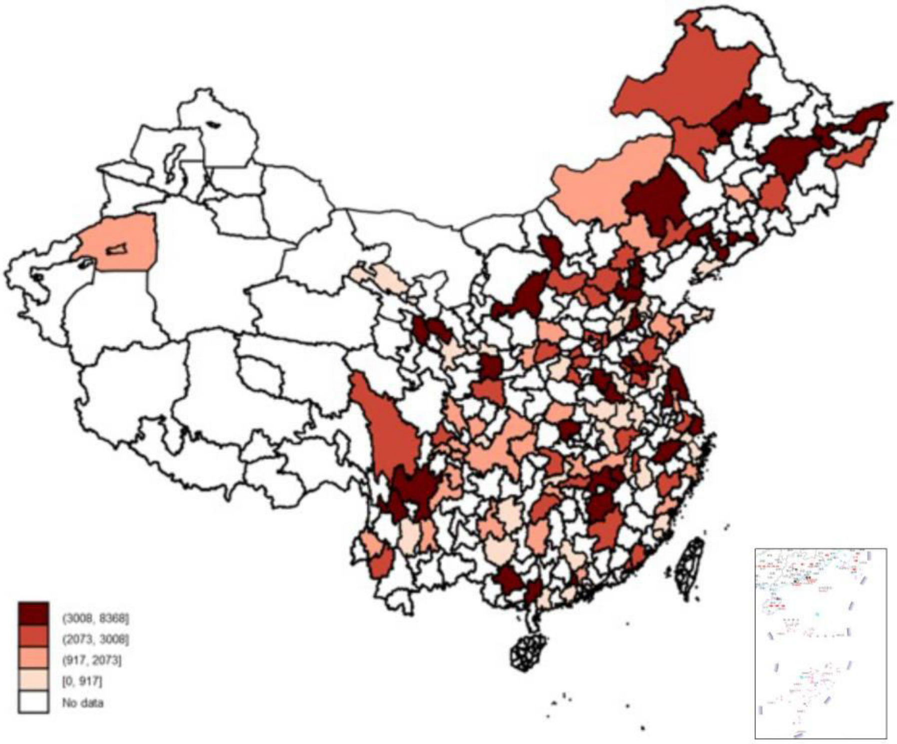

Fig. S6. Expenses of Inpatient Visits of Hypertensive Patients Across PAR in 2015
