# Supplementary material for: Regional variation of medical expenditures attributable to hypertension in China’s middle-aged and elderly population
Source: Medicine (Baltimore). 2022 Dec 23;101(51):e32395. doi: 10.1097/MD.0000000000032395 (PMC9794296; doi:10.1097/MD.0000000000032395)
Supplement: Supplementary file 3 [file medi-101-e32395-s003.pdf]

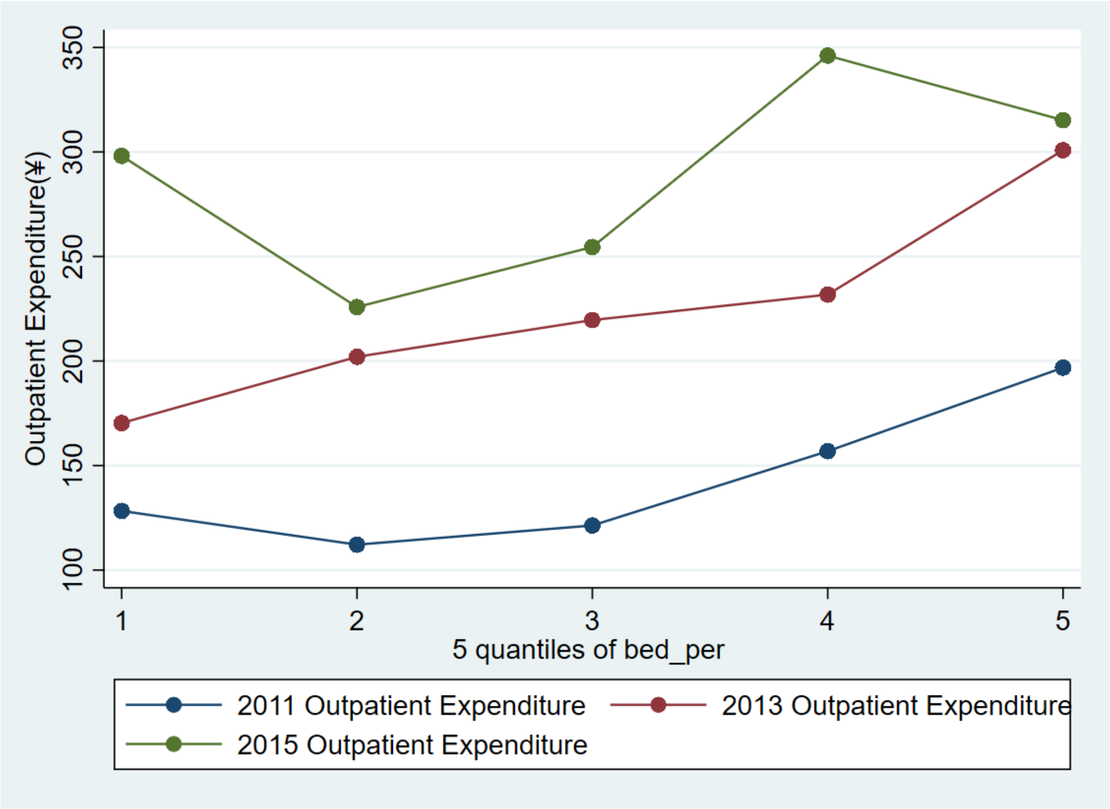

Fig S7. Expenses of Outpatient of Hypertensive Patients Across PAR Bed Pre 10 000 by Year

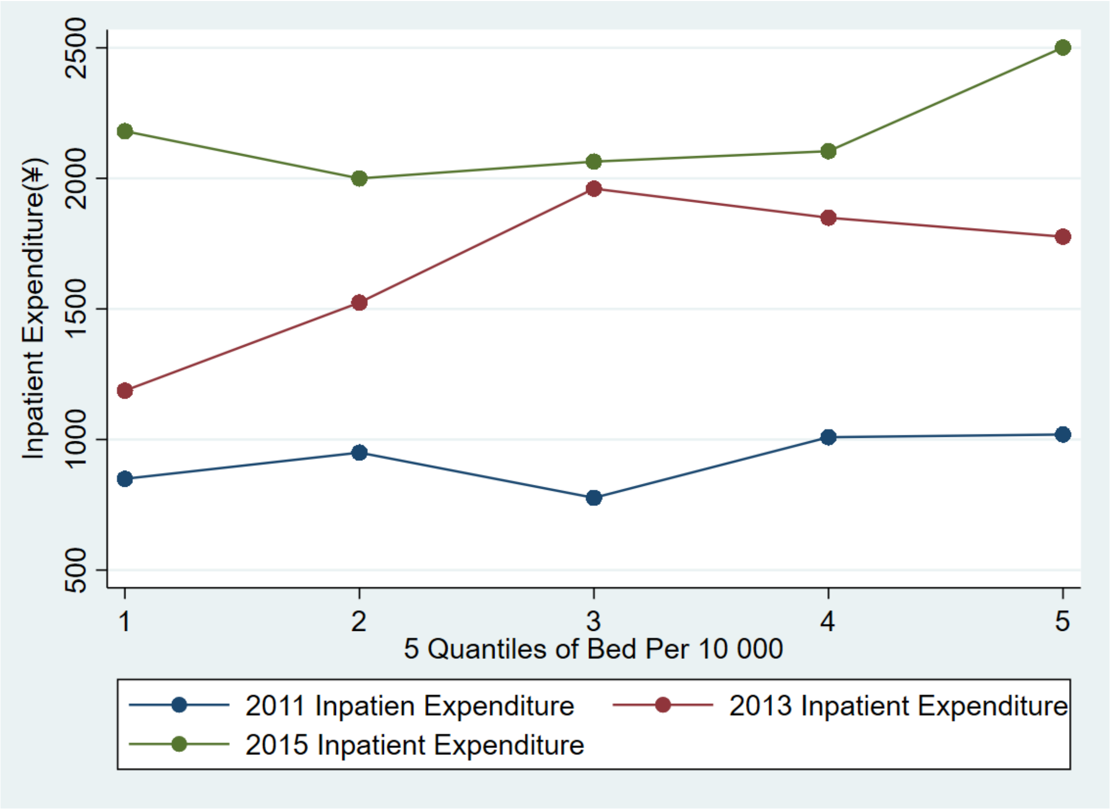

Fig S8. Expenses of Inpatient of Hypertensive Patients Across PAR Bed Pre 10 000 by Year
